# Comprehensive genomic analysis reveals clonal origin and subtype-specific evolution in a case of sporadic multiple meningiomas

**DOI:** 10.1007/s10014-024-00486-9

**Published:** 2024-07-27

**Authors:** Maki Sakaguchi, Masafumi Horie, Yukinobu Ito, Shingo Tanaka, Keishi Mizuguchi, Hiroko Ikeda, Etsuko Kiyokawa, Mitsutoshi Nakada, Daichi Maeda

**Affiliations:** 1 Department of Molecular and Cellular Pathology, Graduate School of Medical Sciences, Kanazawa University, Kanazawa, Ishikawa Japan; 2 Department of Neurosurgery, Graduate School of Medical Sciences, Kanazawa University, Kanazawa, Ishikawa Japan; 3 Department of Diagnostic Pathology, Kanazawa University Hospital, Kanazawa, Ishikawa Japan; 4 Department of Oncologic Pathology, Kanazawa Medical University, Kanazawa, Ishikawa Japan

**Keywords:** Multiple meningiomas, Occult meningioma, Copy number alteration, Whole-exome sequencing

## Abstract

Meningioma is the most common primary intracranial tumor in adults, with up to 10% manifesting as multiple tumors. Data on the genomic and molecular changes in sporadic multiple meningiomas are scarce, leading to ongoing debates regarding their evolutionary processes. A comprehensive genetic analysis of a large number of lesions, including precursor lesions, is necessary to explore these two possible origins: clonal and independent. In the present study, we performed whole-exome sequencing and analyzed somatic single-nucleotide variants (SNVs), insertions/deletions (INDELs), and copy number alterations (CNAs) in a patient with sporadic multiple meningiomas. These meningiomas included two mass-forming lesions of different histological subtypes (transitional and chordoid) and two small meningothelial nests. Genetic analysis revealed CNAs on chromosomes 22q and Y as common abnormalities in the two largest tumors. Furthermore, we identified SNV/INDELs unique to each focus, with *NF2* mutation prevalent in the transitional meningioma and *CREBBP* mutation in the chordoid meningioma. Loss of chromosome 22 was detected in two small meningothelial nests. Overall, we elucidated the clonal origin and subtype-specific evolution of multiple meningiomas in this case. CNAs may serve as the initial driving event in meningioma development.

**Supplementary Information:**

The online version contains supplementary material available at 10.1007/s10014-024-00486-9.

## Introduction

Meningioma is the most common primary intracranial tumor in adults. Its incidence is higher in women than in men but the reason for this difference is unclear [1]. Most meningiomas are solitary; however, up to 10% of cases manifest as multiple tumors [2]. Recent evidence suggests that the incidence of multiple meningiomas may be even higher than 10% [3]. Multiple meningiomas are either sporadic or familial, with some sporadic cases being radiation-induced. The standard treatment for meningioma is surgical resection. In patients with multiple meningiomas, clinical decisions regarding the lesions that should be surgically resected and the order of their resection are often difficult. It is a chronic disease that requires repeated interventions and lifelong surveillance to achieve disease control [4]. In terms of prognosis, patients with multiple meningiomas exhibit shorter overall survival, progression-free survival, and time to second intervention than patients with a solitary meningioma [3, 5]. Notably, a study that involved a large cohort of patients with multiple meningiomas revealed that a greater number of lesions, older age at diagnosis, and male sex were significantly negatively associated with overall survival [3].

Recent advances in the genomic analysis of solitary meningiomas have shed light on the relationships among the histological subtype, site of origin, malignancy, and prognosis [6, 7]. Approximately 50% of meningiomas exhibit *NF2* mutations and/or loss of chromosome 22, where *NF2* is located. These genomic changes have been proven to be associated with atypical clinical and histological presentations due to genomic instability. Specifically, they show a predilection for meningiomas of the cerebrum, cerebellar hemispheres, posterior skull base, and spinal regions as well as transitional and fibrous subtypes [8, 9]. Meningiomas without *NF2* alterations are clinically benign and typically localized to the medial skull base. Their characteristic genomic changes include mutations of *TRAF7*, *KLF4*, *AKT1*, and *SMO*. These mutations occur in a histological subtype-specific manner. Meningothelial and transitional meningiomas frequently harbor *TRAF7* and either *AKT1* or *SMO* mutations [9]. Mutations in *SMARCE1*, *BAP1*, or a combination of *TRAF7* and *KLF4* are associated with clear cell, rhabdoid, or secretory meningioma variants, respectively [9, 10].

Multiple meningiomas are associated with familial tumor syndromes such as neurofibromatosis type 2 and schwannomatosis, which are genetically characterized by germline mutations of *NF2* and *SMARCB1*, respectively [11]. However, data on the genomic and molecular changes in patients with sporadic multiple meningiomas are scarce, leading to ongoing debates regarding their evolutionary processes [12, 13]. Two hypotheses have been proposed to explain the pathogenesis of sporadic multiple meningiomas: a clonal origin and an independent origin. Studies supporting the clonality hypothesis suggest that multiple meningiomas arise from a specific neoplastic clone that proliferates along the meninges to form multifocal lesions [12]. This hypothesis is supported by observations that most sporadic multiple meningiomas exhibit identical histological features. By contrast, some researchers consider multiple meningiomas as independent lesions because some of these tumors exhibit various histological subtypes or grades [14, 15]. A comprehensive genetic analysis of a large number of lesions, including precursor lesions, is necessary to resolve this issue. Although gaining a thorough understanding of meningiomas development requires the examination of precursor lesions such as small meningothelial nests, no reports to date have discussed the genomic changes in the putative precursor of mass-forming meningioma.

In the present study, we performed whole-exome sequencing (WES) and analyzed somatic single-nucleotide variants (SNVs), insertions/deletions (INDELs), and copy number alterations (CNAs) in a patient with sporadic multiple meningiomas. The meningiomas comprised two mass-forming lesions of different histological subtypes (transitional and chordoid) and two small meningothelial nests. The clonality and evolutional processes of these lesions were analyzed to elucidate the pathogenesis of sporadic multiple meningiomas.

### Clinical summary

**Fig. 1:**
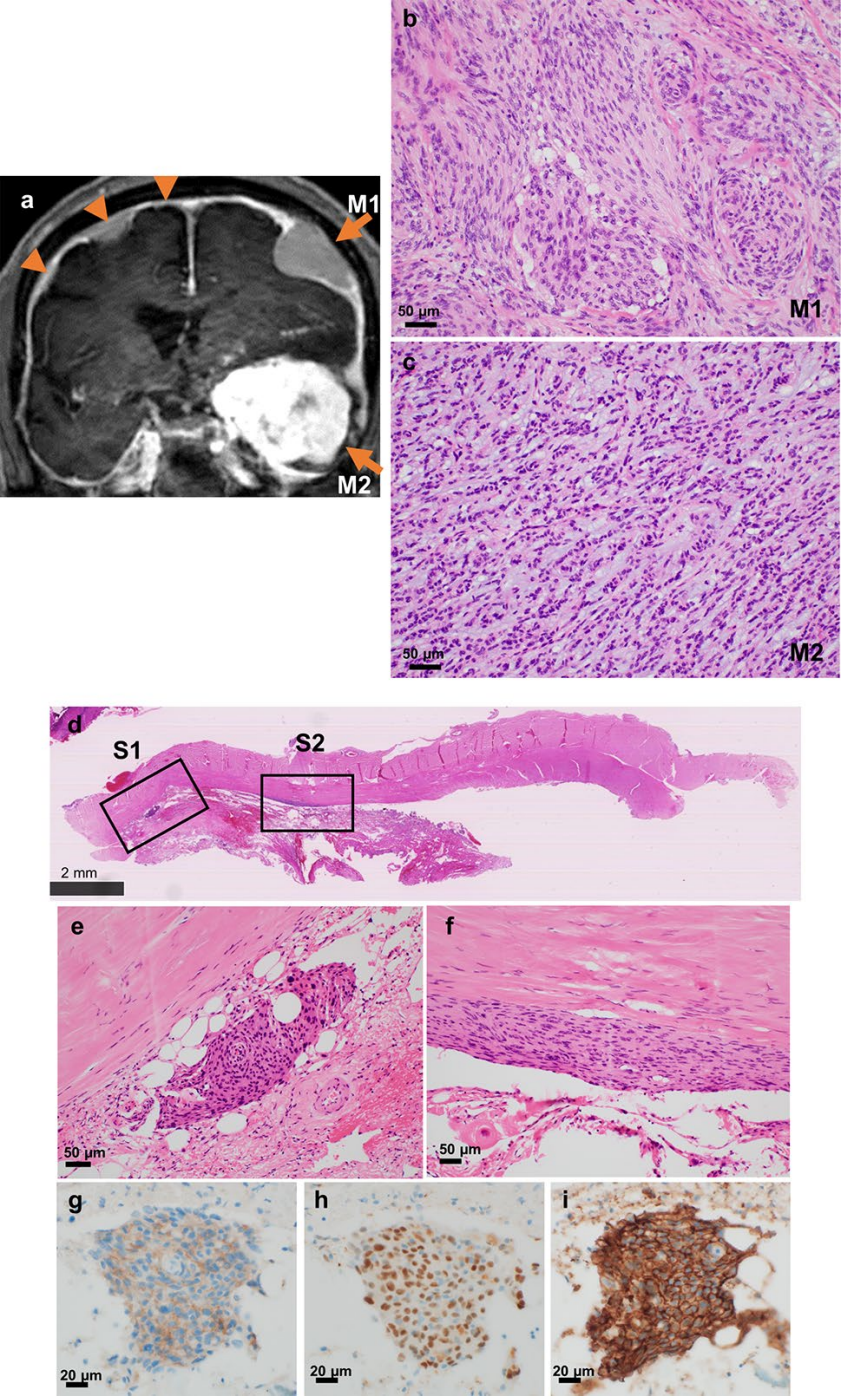
**a** Gadolinium enhanced T1-weighted magnetic resonance image and **b**–**i** histological images of the patient in the present case. **a**–**c** Mass-forming tumors and **d**–**i** non-mass forming precursor lesions. In addition to the multiple lesions in the bilateral convexities, **a** a large tumor with strong homogenous gadolinium enhancement was present in the left sphenoidal ridge. The two large tumors located in the left convexity (M1) and on the left sphenoidal ridge (M2) were resected. Arrowhead: non-resected multiple lesions in the convexity; arrow: resected tumors. Histologically, **b** M1 was composed of proliferative meningothelial cells arranged in bundles or whorls, whereas **c** M2 exhibited cord-like arrays of epithelioid tumor cells within an abundant basophilic myxoid matrix. **d** Low magnification of the two microdissected areas of the dura surrounding the left convexity tumor. **e**, **f** Microscopically, the small meningothelial nests (S1, S2) composed of more than 10 layers of oval or spindle-shaped meningothelial cells with or without psammoma bodies were identified. S1 and S2 were 2.7 mm apart. In immunohistochemistry, the small meningothelial nests are positive for EMA **(g)**, PgR **(h)**, and SSTR2a **(i)**

An 83 year-old man was incidentally discovered to have bilateral frontal convexity tumors, measuring 9 mm on the right and 16 mm on the left, 9 years prior to presentation. These asymptomatic tumors were monitored over time and identified as meningiomas through imaging (supplementary Fig. 1). The patient had no familial history of the disease and no evidence of neurofibromatosis. He subsequently developed cognitive dysfunction coinciding with the identification of a new lesion on the left sphenoidal ridge. Preoperative magnetic resonance (MR) imaging revealed that this was the largest tumor, measuring 50 mm, and it exhibited strong homogenous enhancement (Fig. 1a). In addition, seven other smaller tumors had newly appeared on the images (supplementary Fig. 2). The previously existing bilateral frontal convexity tumors were mildly enlarged, measuring 18 mm on the right and 24 mm on the left.

### Pathological and genetic findings

We simultaneously resected two largest mass-forming meningiomas, one in the left convexity (M1) and one on the left sphenoidal ridge (M2), along with the left convexity dura, which contained two non-mass-forming small meningothelial nests (S1, S2). Gross total resection was performed on M1 and M2, and all tissue fragments up to 10 mm for M1 and up to 25 mm for M2 were submitted for histological examination. M1 was a transitional meningioma composed of proliferative meningothelial cells arranged in bundles or whorls (Fig. 1b). M2 was a chordoid meningioma consisting of cord-like arrays of epithelioid cells within an abundant basophilic myxoid matrix (Fig. 1c). Both lesions did not contain components composed of other subtypes. M1 and M2 were grade 1 and 2 tumors, respectively, according to the 2021 World Health Organization (WHO) classification. In the dura surrounding the left convexity tumor, scattered small meningothelial nests composed of more than 10 layers of oval or spindle-shaped meningothelial cells with or without psammoma bodies were observed, and S1 and S2 were 2.7 mm apart (Fig. 1d–g). Immunohistochemistry showed that M1 and M2 were diffusely positive for epithelial membrane antigen (EMA), progesterone receptor (PgR), and somatostatin receptor 2a (SSTR2a), and that M2 was negative for brachyury. Ki-67 labeling index were 0.8% and 0.6%, respectively. The small meningothelial nests were diffusely positive for EMA and SSTR2a, and partially positive for PgR. The primary antibodies used in the immunohistochemical analysis are listed in Supplementary Table 1.

**Fig. 2:**
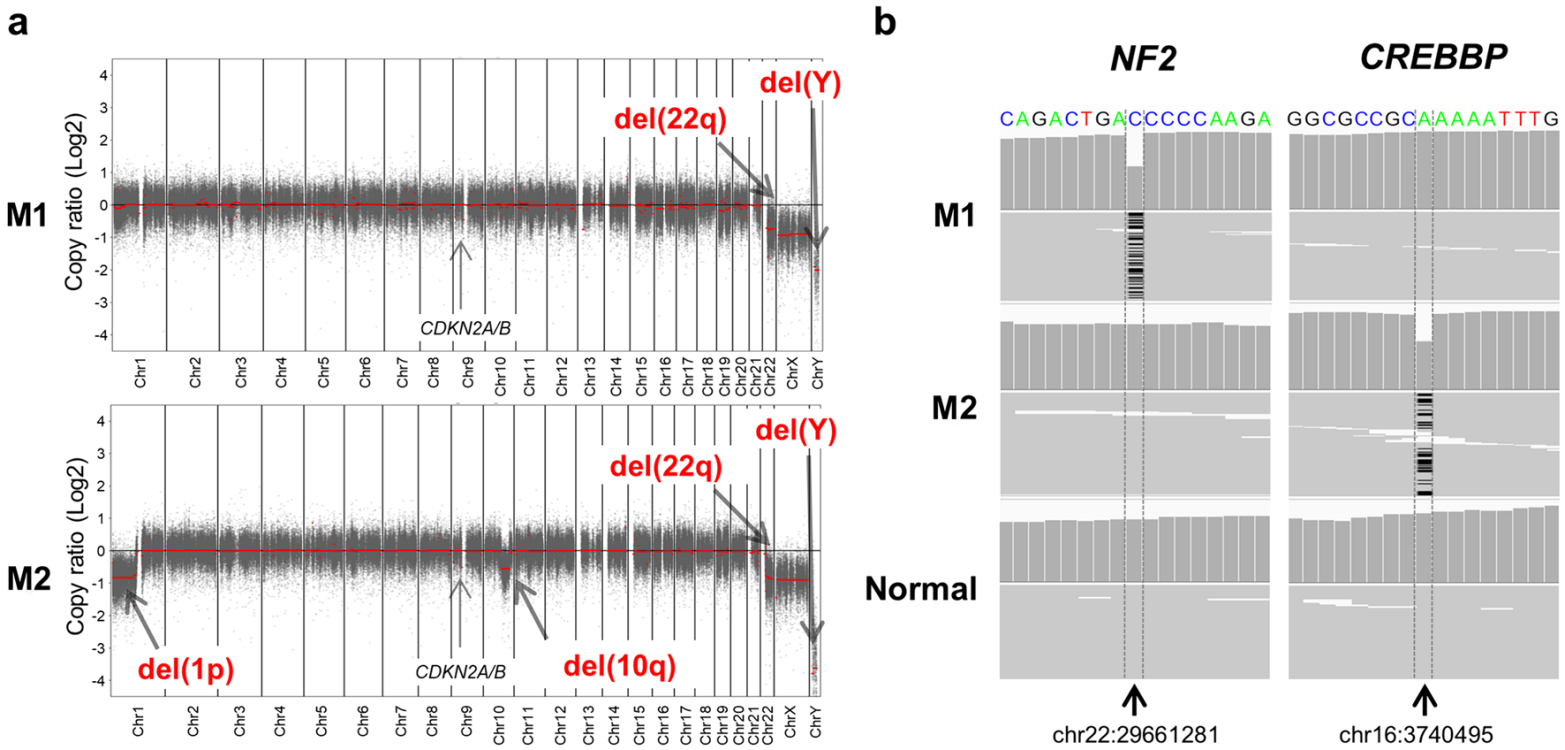
**a** Copy number alterations and **b** deletion mutations of M1 and M2 detected by whole-exome sequencing. **a** M1 showed loss of chromosomes 22q and Y, whereas M2 exhibited loss of chromosomes 1p, 10q, 22q, and Y. Homozygous deletions of *CDKN2A* and *CDKN2B* were not detected in either M1 or M2. **b** Information and coordinates of genetic mutations observed in M1 and M2. The *NF2* frameshift mutation (c.503delC:p.K170Rfs*43) in M1 and *CREBBP* frameshift mutation (c.3923delT:p.L1308Cfs*30) in M2 were considered significant

**Fig. 3:**
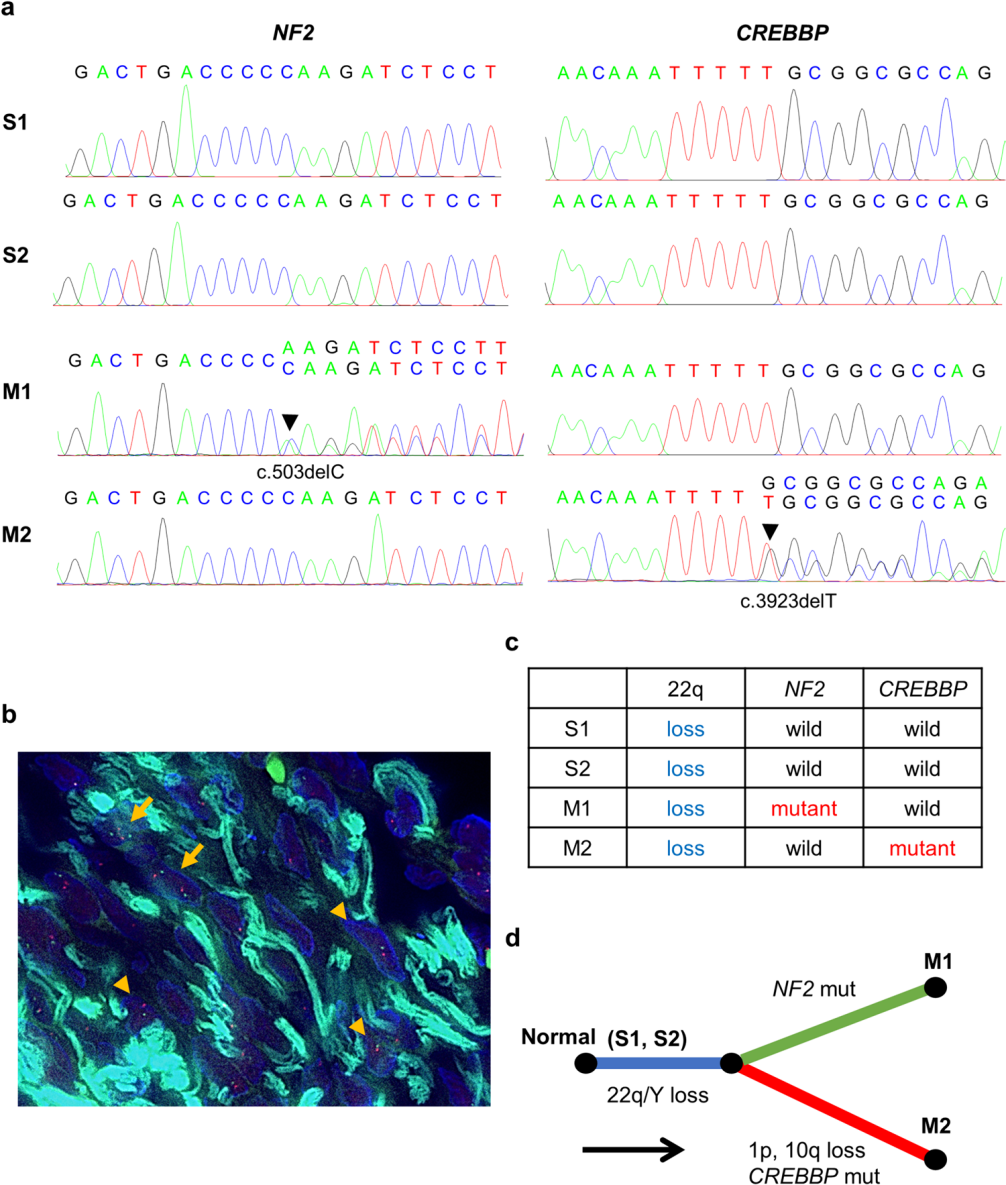
Mutation and copy number analyses of small meningothelial nests by Sanger sequencing and fluorescence *in situ* hybridization of chromosome 22. **a** The *NF2* frameshift mutation in M1 and *CREBBP* frameshift mutation in M2 were confirmed by Sanger sequencing. These mutations were not found in S1 and S2. **b** Two small meningothelial nests (S1, S2) contained scattered cells exhibiting monosomy of chromosome 22q with one red and one green signal (arrowhead: heterozygous deletion; arrow: non-deleted). **c** Summary of CNA of chromosome 22 and mutations of *NF2* and *CREBBP* in two small meningothelial nests and two tumors. **d** Phylogeny inferred from the somatic CNA and SNV/INDEL

WES was conducted to assess the clonality of the two resected meningiomas. Genomic DNA was extracted from frozen specimens of M1, M2 and background normal brain tissue. Libraries were prepared for each sample using a SureSelect Human All Exon V6 kit (Agilent Technologies) in accordance with the manufacturer’s recommendations. CNA analysis indicated loss of chromosomes 22q and Y in M1 and loss of chromosomes 1p, 10q, 22q, and Y in M2 (Fig. 2a). Thus, loss of chromosomes 22q and Y were common events in both M1 and M2. Mutational analysis revealed 52 and 66 SNVs/INDELs in M1 and M2, respectively. After the application of strict filtering criteria, 14 mutations were retained in each tumor, with no overlapping mutations between M1 and M2 (Table 1). Among these genes, *NF2* frameshift mutation (c.503delC:p.K170Rfs*43) in M1 and *CREBBP* frameshift mutation (c.3923delT:p.L1308Cfs*30) in M2 were highlighted upon comparison with previously published data on genes mutated in at least two cases of meningiomas (Fig. 2b) [16]. Sanger sequencing was performed to validate the identified gene mutations, confirming the mutations of *NF2* in M1 and *CREBBP* in M2 (Fig. 3a). Homozygous deletions of *CDKN2A* and *CDKN2B*, as well as *TERT* promoter hotspot mutations that are indicative of WHO grade 3 tumors, were not detected in either M1 or M2 by WES and Sanger sequencing (data not shown).

**Table 1:** Nonsynonymous gene mutations from the transitional meningioma (M1) and chordoid meningioma (M2) detected by whole-exome sequencing^*^

Gene	Mutation type	Nucleotide change	Amino acid change	Allele frequency (%)
Transitional meningioma (M1)				
REPS2	Missense	c.G650A	p.S217N	74.3
NF2	Frameshift	c.503delC	p.K170Rfs*43	44.8
OXCT1	Missense	c.G296A	p.R99Q	43.8
ZNF469	Missense	c.C11599G	p.Q3867E	40.7
COL4A2	Missense	c.C3076T	p.P1026S	38.5
GLI4	Missense	c.G283A	p.G95R	38.4
TRPA1	Missense	c.T1448C	p.M483T	38.1
SOS1	Missense	c.C3589G	p.P1197A	34.7
PPP1R7	Missense	c.G907C	p.A303P	34.5
CLCN2	Missense	c.C2096T	p.S699F	34.5
DNAH5	Stop-gain	c.C6763T	p.R2255X	34.2
COL16A1	Missense	c.C162G	p.154 M	33.1
CYB5R4	Missense	c.G923T	p.G308V	24.4
DNAH12	Missense	c.A2274C	p.K758N	20.8
Chordoid meningioma (M2)				
AHDC1	Missense	c.G1760A	p.R587Q	57.1
TYK2	Missense	c.C2315T	p.P1985fs	50.5
TNXB	Frameshift	c.5955delC	p.P1985fs	48.0
SUCO	Missense	c.G530A	p.S177N	44.5
DPH7	Missense	c.C349T	p.R117W	44.3
SUN1	Stop-gain	c.G1638A	p.W546X	44.2
NLRC3	Missense	c.C1340T	p.S447L	43.5
ZNF280D	Missense	c.C359T	p.S120L	42.7
SLMAP	Stop-gain	c.C61T	p.Q21X	41.9
NCOA6	Missense	c.T3095C	p.1032A	40.4
MYH7	Missense	c.A3536G	p.E1179G	39.8
CREBBP	Frameshift	c.3923delT	p.L1308Cfc*30	37.3
TLR9	Missense	c.A2432G	p.D811G	30.6
RNF208	Stop-gain	c.G198A	p.V22M	29.5

*The filtering steps were performed according to the following four parameters: gnomad_AF_popmax < 0.01, normal VAF (NVAF) < 0.05, tumor VAF (TVAF) > 0.2, and TVAF/NVAF > 5.0

Finally, to elucidate the clonal origin and subtype-specific evolution of multiple meningiomas, Sanger sequencing and Fluorescence* in situ* hybridization (FISH) of S1 and S2 were performed, and the gene mutations and CNAs were compared to those of M1 and M2. S1 and S2 of the dura mater were microdissected from sections stained with hematoxylin and eosin (HE) for DNA extraction. Sanger sequencing was performed to evaluate the gene mutations found in M1 and M2. Sequencing showed no *NF2* or *CREBBP* mutations in S1 and S2. The primers used in the Sanger sequencing are listed in Supplementary Table 2. Furthermore, FISH was performed to evaluate the copy number of chromosome 22 in the S1 and S2 using an *NF2* (22q12) deletion probe (Guang Zhou LBP Medicine Science & Technology, Guangzhou, China). Each lesion was compared to a section stained with HE to aid the identification of proliferative meningeal cells. After identifying an internal control, such as endothelial cells or lymphocytes, the number of nuclei was counted in at least 30 cells. The criteria for determining the presence of loss of chromosome 22 was defined as the presence of ≥ 4.2% deleted cells based on a previous study using age- and sex-matched normal control bone marrow samples [17]. FISH of chromosome 22 in the S1 and S2 revealed scattered cells exhibiting monosomy of chromosome 22q with one red and one green signal (Fig. 3b). The percentages of the meningeal cells displaying deletions in each lesion were 67.1% and 40.9%, respectively. These lesions were determined to have a loss of chromosome 22q. Collectively, they were considered to represent the clonal origin with loss of chromosome 22q, which then underwent subtype-specific evolution and acquired *NF2* and *CREBBP* mutations (Fig. 3c, d).

## Discussion

We performed WES of sporadic multiple meningiomas of different histological subtypes and identified CNAs on chromosomes 22q and Y as common genetic abnormalities. SNVs/INDELs unique to each focus were also detected. All of the lesions, including small meningothelial nests, were considered to be associated with *NF2* based on the loss of chromosome 22q. Interestingly, the transitional meningioma (M1) had a second hit of *NF2* and the chordoid meningioma (M2) had a *CREBBP* mutation. *CREBBP* is a chromatin-remodeling gene that is more enriched in chordoid than non-chordoid meningiomas [18]. This difference indicates that an epigenetic abnormality caused by *CREBBP* mutation may induce chordoid change against a background of chromosome 22 loss. The multiple meningiomas in this study were thought to be of clonal origin with subtype-specific evolution, although independent origin could not be completely ruled out. A WES analysis of multiple meningiomas demonstrated clonal origin in five of six cases and subsequent branched evolution that resulted in inter-tumoral heterogeneity represented by different histologic subtypes and grades [13]. Other studies of multiple meningiomas of different histological subtypes have suggested that each lesion develops independently, but these studies did not evaluate CNAs and may not have accurately determined clonality [15, 19]. Accurate assessment of the clonality of multiple meningiomas requires not only mutational analysis but also CNA analysis. Because multiple meningiomas may be both clonal and independent in origin, a large study is necessary.

Our study is the first to analyze genetic changes in precursor lesions of mass-forming meningiomas. Loss of chromosome 22 was detected in two small meningothelial nests, whereas the somatic mutations in *NF2* and *CREBBP* found in the mass-forming tumor were not present. It is often difficult to determine whether microscopic proliferative lesions consisting of meningothelial cells are reactive or neoplastic simply by observing their morphology. Perry et al. performed FISH in 11 cases of meningothelial hyperplasia and found no case of deletion of chromosome 22 [20]. The presence of the CNA in our patient indicated that the small meningothelial nests were neoplastic. This hypothesis is supported by immunohistochemical results showing that EMA, PgR, and SSTR2a, which do not stain in normal meninges, were positive in the small meningothelial nests [21]. The presence of CNAs has been reported in precancerous lesions such as intestinal metaplasia (which is a risk factor for gastric cancer) and clonal hematopoiesis (which is implicated in the development of hematological malignancies) [22, 23]. Our findings indicate that CNAs are linked to the development of meningioma and that they precede SNVs/INDELs as genetic abnormalities, in line with a previous study of multiple meningiomas [13].

Chromosome 22q deletion is identified in approximately 50% of meningiomas, marking this as a critical early event in the onset of *NF2*-related meningiomas [24, 25]. Our study suggests that loss of chromosome Y (LOY) is also an initial event in the development of meningioma, concurrent with the loss of chromosome 22. However, the clinical and biological significance of the Y chromosome in meningioma remains largely unexplored. To date, only a few studies that have used FISH analysis of meningiomas have shown that LOY in men represents the second most frequent aberration, accounting for 28% to 46% of cases, following loss of chromosome 22 [17, 26]. Qi et al. compiled an extensive catalog of LOY across more than 5000 primary tumors from men in The Cancer Genome Atlas, demonstrating that LOY is exceedingly prevalent in numerous tumor types and suggesting its potential driving role in uveal melanoma [27]. Furthermore, LOY is associated with adverse outcomes in patients with bladder cancer, and cancer cells exhibiting LOY have been shown to modify T-cell functionality, leading to exhaustion of CD8 + T cells in the tumor microenvironment and increasing their susceptibility to PD-1-targeted immunotherapy [28]. In the context of meningioma, LOY may play a role in the tumorigenesis of meningiomas in men, potentially contributing to the poorer prognosis in men than in women.

## Conclusion

Genomic analysis of sporadic multiple meningiomas of different histological subtypes revealed the clonal origin and subtype-specific evolution. CNAs may serve as the initial driving event in meningioma development. Further investigation involving a larger cohort is warranted.

## Supplementary Information

Below is the link to the electronic supplementary material.

Supplementary file1 (DOCX 1898 KB)

## Data Availability

WES data can be obtained from the corresponding authors upon reasonable request.
