# Supplementary material for: Comprehensive genomic analysis reveals clonal origin and subtype-specific evolution in a case of sporadic multiple meningiomas
Source: Brain Tumor Pathol. 2024 Jul 27;41(3-4):132–8. doi: 10.1007/s10014-024-00486-9 (PMC11499534; doi:10.1007/s10014-024-00486-9)
Supplement: Supplementary file 1 — Supplementary file1 (DOCX 1898 KB) [file 10014_2024_486_MOESM1_ESM.docx]

**Supplementary information**

**Comprehensive genomic analysis reveals clonal origin and subtype-specific evolution in a case of sporadic multiple meningiomas**

Maki Sakaguchi^1^, Masafumi Horie^1*^, Yukinobu Ito^1^, Shingo Tanaka^2^, Keishi Mizuguchi^3^, Hiroko Ikeda^3^, Etsuko Kiyokawa^4^, Mitsutoshi Nakada^2^, Daichi Maeda^1^

*Corresponding author

Masafumi Horie, MD, PhD

Department of Molecular and Cellular Pathology, Graduate school of Medicine, Kanazawa University.

Takara Machi 13-1, Kanazawa City, Ishikawa, Japan

E-mail: mhorie@med.kanazawa-u.ac.jp

Phone: +81-76-265-2192

FAX: +81-76-234-4228

**Supplementary Figure 1.**

**Progression of tumors by gadolinium-enhanced T1-weighted magnetic resonance imaging (MRI).**

**9 years before 8 years before Preoperative**


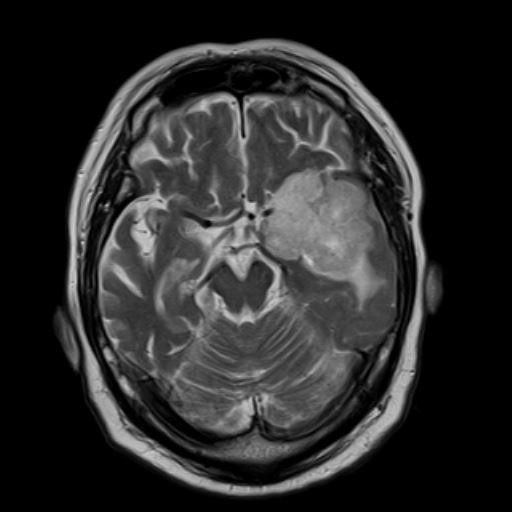

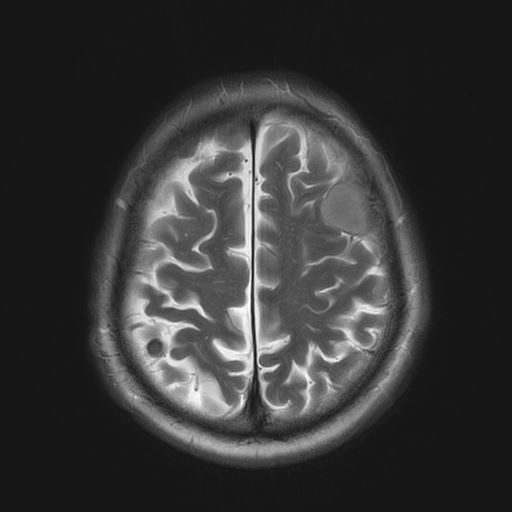

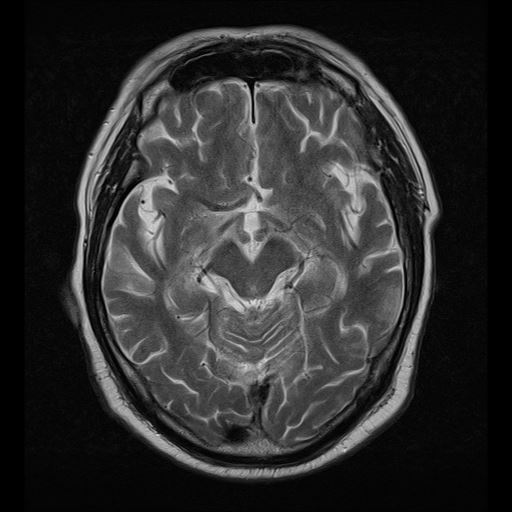

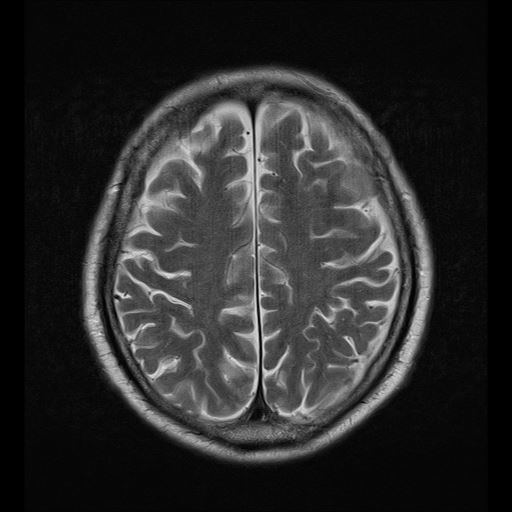

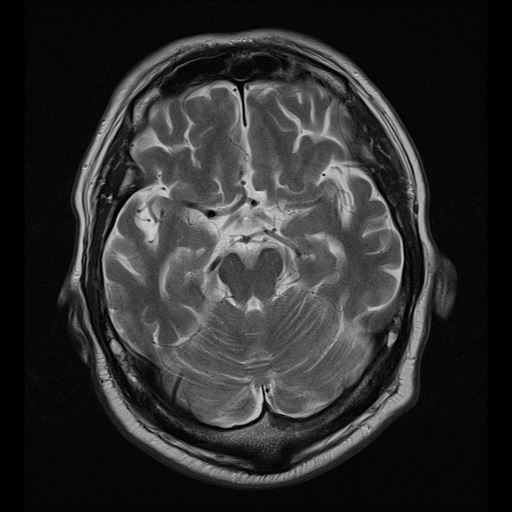

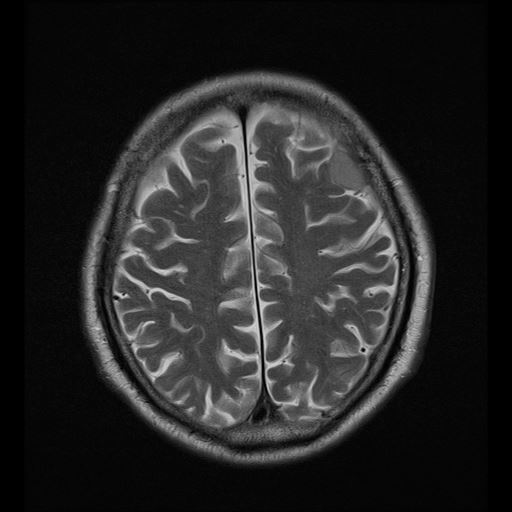


The left frontal convexity tumor (M1) had grown slowly. In contrast, the largest tumor on the left sphenoidal ridge (M2) was not recognized until 8 years before surgery and grew at a faster rate. Arrow: left frontal convexity tumor (M1); Arrowhead: left sphenoidal ridge tumor (M2).

**Supplementary Figure 2.**

**Horizontal cross-sections of preoperative MRI.**


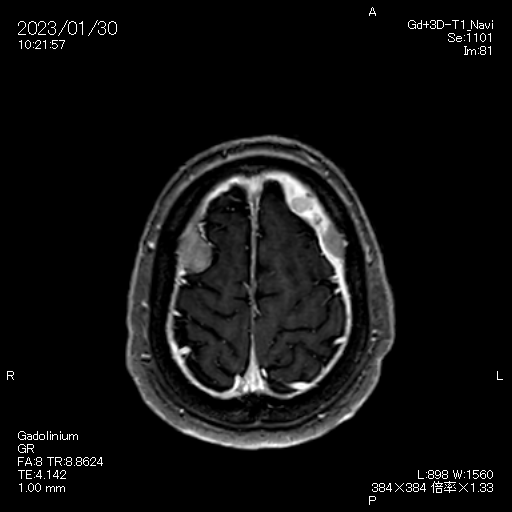


**18mm**

**7mm**

**M1**


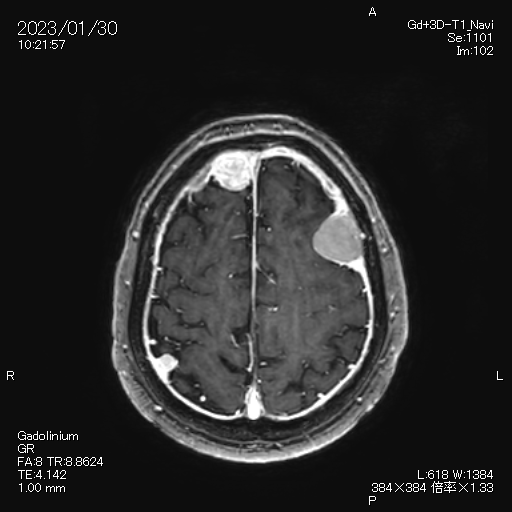


**24mm**

**19mm**

**10mm**

**M1**


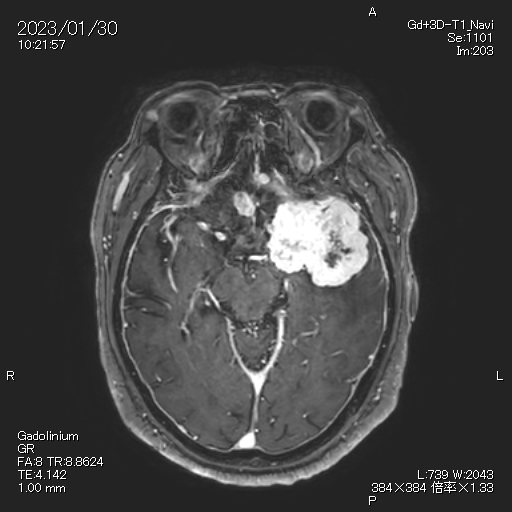


**50mm**

**4mm**

**12mm**

**M2**

Multiple tumors and their respective size are shown. In addition to the three previously known tumors (arrowhead) in the bilateral convexity, seven new tumors (arrow) had been appeared, including the largest tumor on the left sphenoidal ridge.


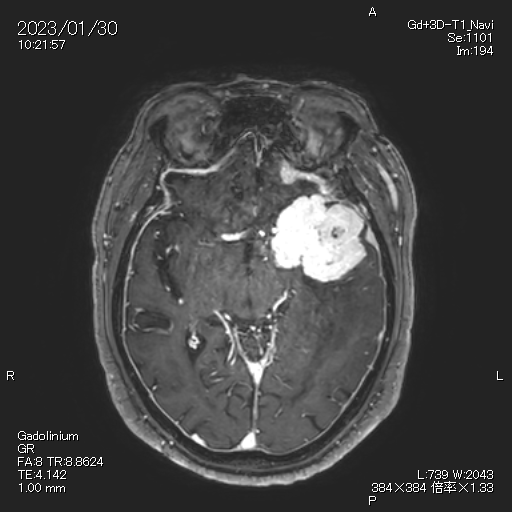


**5mm**

**7mm**

**M2**

**Supplementary Table 1.** List of antibodies used in this study.

| **Antibody (clone)** | **Source** | **Dilution** |
| --- | --- | --- |
| EMA (E2) | Dako/Agilent Technologies, Santa Clara, CA, USA | Ready to use |
| PgR (1E2) | F. Hoffmann-La Roche, Basel, Switzerland | Ready to use |
| SSTR2a (EP149) | Nichirei | Ready to use |
| brachyury (1H9A2) | Abcam, Cambridge, UK | 1:400 |
| Ki-67 (30-9) | F. Hoffmann-La Roche, Basel, Switzerland | Ready to use |

**Supplementary Table 2.** List of primers for Sanger sequencing used in this study.

| **Primer** | **Sequence** |
| --- | --- |
| *NF2* forward | 5′-TCAGCTGGCGCTTACAGTAG-3′ |
| *NF2* reverse | 5′-GGGAAAGATCTGCTGGACCC-3′ |
| *CREBBP* forward | 5′-GACCTCCACCGTCTTGTCTG-3′ |
| *CREBBP* reverse | 5′-TCTCACCTGCTCCTTCTGGA-3′ |
| *TERT* forward | 5′-GTCCTGCCCCTTCACCTT-3′ |
| *TERT* reverse | 5′-CAGCGCTGCCTGAAACTC-3′ |
